# Effects of Household Cooking on Mineral Composition and Retention in Widespread Italian Vegetables

**DOI:** 10.3390/nu17030423

**Published:** 2025-01-24

**Authors:** Silvia Lisciani, Altero Aguzzi, Paolo Gabrielli, Emanuela Camilli, Loretta Gambelli, Luisa Marletta, Stefania Marconi

**Affiliations:** Council for Agricultural Research and Economics, Research Centre for Food and Nutrition, via Ardeatina 546, 00178 Rome, Italy; altero.aguzzi@crea.gov.it (A.A.); paolo.gabrielli@crea.gov.it (P.G.); emanuela.camilli@crea.gov.it (E.C.); loretta.gambelli@crea.gov.it (L.G.); luisa.francesca.marletta@gmail.com (L.M.); stefania.marconi@crea.gov.it (S.M.)

**Keywords:** cooking methods, retention factors, minerals, nutrient composition

## Abstract

Background/Objectives: The process of cooking food can result in alterations to its nutrient composition due to changes in water content and the destruction or loss of certain micronutrients that occur in response to heat. This study examined the impact of diverse cooking techniques, namely grilling, microwave, and steam, on the macronutrients and minerals of vegetables commonly utilized in Italian cuisine (two varieties of zucchini, eggplants, and potatoes). Methods: The proximate composition was determined according to the Association of Official Analytical Chemists (AOAC) methods. The content of the minerals (Ca, K, P, Mg, Na, Fe, Zn, and Mn) was determined via ICP plasma after liquid washing. Results: Regarding macronutrients, the results revealed a notable difference in the carbohydrate profiles, whereas mineral retention demonstrated considerable heterogeneity. Some minerals, such as Na, Ca, Mn, and Fe, were found to be more prone to significant increases or losses. Moreover, the true retention factor (TR) calculations indicated that microwave cooking resulted in higher retention compared to the other methods for zucchini, while grilling demonstrated higher TR than microwave cooking for eggplants. Potatoes exhibited lower TR values than the other vegetables and their steaming resulted in higher retention than microwave cooking for K, P, Fe, and Zn. Conclusions: The results confirm the heterogeneous behaviors of minerals in commonly consumed Italian vegetables subjected to different cooking methods. The data underscore the need for additional research to understand the effects of heat treatments on mineral profiles and to determine specific retention factors linked to various cooking techniques. The significant gap between “true” and “apparent” retention factors, caused by changes in water content during cooking, highlight the need for new experimental data to update and enrich the existing literature on this topic.

## 1. Introduction

The consumption of vegetables is linked to the promotion of a balanced diet and a decrease in non-communicable disease risk, attributed to the synergistic benefits of dietary fiber, vitamins, minerals, and antioxidants [1,2,3]. Most vegetables can be prepared in the domestic setting by removing inedible portions, washing, cutting, and cooking using several methods including boiling, frying, roasting, steaming, and grilling [4,5,6,7].

Cooking techniques can enhance food safety and organoleptic properties, while also affecting nutrient concentration and bioavailability in vegetables [8,9]. Traditional methods like stewing and boiling may lead to nutrient loss, mainly of antioxidants and water-soluble vitamins [5,7,10,11,12]. Methods such as microwaving, steaming and sous-vide cooking offer advantages in nutrient retention [13,14], but numerous studies demonstrate conflicting results regarding how cooking affects nutrient stability [6,15]. The effectiveness of cooking methods also varies depending on the food properties and preparation techniques, complicating advice on optimal cooking for nutrient preservation [16,17,18,19,20,21,22].

Minerals maintain their stability during heat treatment and are less susceptible to damage than vitamins; however, they may leach into the cooking medium to an extent that depends on the structure, size, and type of the food, as well as the preparation, cooking method, and time used [16,23,24].

For example, the process of cutting vegetables into smaller pieces can facilitate the leaching of nutrients due to the increased surface area, and peeling can result in the loss of minerals that are concentrated near or in the skin [25,26,27]. As regards cooking methods, the application of moist heat treatments which utilize water or other liquids, such as boiling, stewing, braising, and steaming, has been observed to facilitate the loss of minerals [28]. Conversely, dry heat treatments that do not involve water or employ brief cooking times can help in the preservation of minerals [29].

Retention is a measure of the quantity of nutrients retained in the food after preparation, calculated as a proportion of the initial nutrient content. It can be estimated using retention factors, which are particularly useful for vitamins and minerals [30,31,32]. Nutrient retention enables the correction of food composition for changes that occur during the thermal process and can be utilized to identify the most effective method for retaining nutrients, facilitating the assessment of nutrient intake [16,23,32,33]. The retention of micronutrients in cooked foods can be calculated as either apparent retention (AR), based on the dry form, or true retention (TR), based on the fresh form [7,33,34]. AR does not consider the changes in dry matter that occur during processing, whereas TR measures the proportion of a nutrient that remains in the cooked food in relation to the amount of the nutrient present in the food before cooking, providing a more accurate estimate of nutrient retention [32,33,34].

The TR of nutrients in cooked foods differs from the AR depending on the moisture loss from raw or cooked foods to their dried forms; therefore, the AR method is useful when dry matter data are unavailable. It is acknowledged that monitoring weights may not always be a possible option in studies involving industrial processing, but this should be a priority in research examining the cooking of foods [35].

A substantial body of research has examined the impact of cooking methods on the nutritional composition of vegetables, with a particular focus on vitamins and antioxidants, while there is a lack of knowledge regarding the effects of cooking on mineral content.

This study aims to evaluate the macronutrient and mineral composition of vegetables commonly used in Italian and Mediterranean cuisine—zucchini, eggplants, potatoes—before and after using different home cooking methods. To elucidate the differences between the apparent and true retention of minerals, both AR and TR were calculated for various minerals. Thus, it is possible to provide information to consumers on the most suitable cooking methods to preserve the beneficial nutritional characteristics of some of the most consumed vegetables among the Italian population.

## 2. Materials and Methods

### 2.1. Materials

The following three kinds of vegetables commonly used in Italian cuisine were selected for this study: zucchinis (Cucurbita pepo), eggplants (Solanum melongena), and potatoes (Solanum tuberosum). As previously reported in our publication [36], the vegetables were purchased from various supermarkets, markets, and greengrocers in the Rome city area and combined into a composite pool. For eggplants and potatoes, a sample was created by combining different varieties. On the contrary, due to the noticeable difference in the texture of the outer layer, the two varieties of zucchini were studied separately. The vegetables were prepared using common utensils and procedures, including washing and cutting. Only the potatoes were peeled before cooking.

Reagents, enzymes, and standards were purchased from Sigma-Aldrich Srl (Milan, Italy), Carlo Erba (Milan, Italy), J.T. Baker (Deventer, Holland), Megazyme International (Wicklow, Ireland) and BDH Laboratory Supplies (Poole, UK). Double-distilled water (Millipore, Milan, Italy), purified with a Milli-Q™ system, was used throughout the study.

### 2.2. Cooking Methods

Based on consumption habits and product characteristics, different cooking methods were applied to each vegetable as follows (Table 1):

- Steaming was carried out using an electric steamer for home use;
- Microwave cooking was conducted at a power of 700 Watts;
- Grilling was performed using a non-stick pan with a stone base.

All food preparation and cooking steps were carried out in a dedicated experimental kitchen.

The cooking water, used for steaming and microwave cooking, was collected from tap water, which was chosen to reflect real-life home food preparation. In this regard, tap water was characterized by its mineral composition, as reported in Table 2.

After cooking, the samples were drained (if necessary) and weighed after cooling to room temperature [36]. A small amount of raw and cooked samples was immediately used to measure the water content, while the remaining part was homogenized, frozen at −30 °C and freeze-dried for subsequent analyses.

### 2.3. Chemical Analyses

#### 2.3.1. Proximate Composition

All the analyses were carried out according to the Association of Official Analytical Chemists (AOAC) methods [37]. Moisture was determined gravimetrically by drying the samples at 105 °C. Protein was analyzed with the Kjeldahl method using the conversion factor 6.25. Total lipid content was determined after extraction by petroleum, ether using a Soxtec™ 2055 extraction system (Foss, Denmark) according to AOAC Official Method 991.36 [38]. Ash content was determined by incineration in a muffle furnace at 550 °C for 24 h. Total dietary fiber was analyzed using the enzymatic-gravimetric method [39], using a Fibertec™ System E 1023 filtration module (Foss, Denmark). The total starch content was determined using the Megazyme Total Starch Assay kit based on the McClearly method with a standard control “Regular Maize Starch” [40].

The soluble sugars, fructose, and sucrose were extracted with distilled water (20 min using a sonicator) and analyzed by the anion exchange chromatography technique using a DIONEX ion system (mod ICS 5000, Sunnyvale, CA, USA) equipped with a GP50 gradient pump, amperometric detector (HPAE-PAD), and column Carbopac PA1. A solution of 160 mM NaOH was used as the eluent at a flow rate of 1 mL/min. Certified material LGC-7103 was analyzed as the control for the accuracy of the detection of soluble sugars.

#### 2.3.2. Analysis of Minerals

All the samples were analyzed for their content of sodium (Na), potassium (K), phosphorus (P), calcium (Ca), magnesium (Mg), iron (Fe), zinc (Zn), and manganese (Mn). Freeze-dried samples (500 mg) were digested with 6 mL of HNO_3_ and 1 mL of 30% H_2_O_2_ in a 1500-Watt microwave (MLS1200 MEGA Milestone™ Srl, Milan, Italy) with the following heating program: 40 min at 200 °C and then 20 min cooling time. The digested samples were diluted with milliQ water to 10 mL. The mineral concentrations were estimated using Inductively Coupled Plasma-Optical Emission Spectroscopy (ICP-OES) operating in the conditions and parameters described in Table 3.

The accuracy of the method was verified using the following two standard reference materials: spinach (NIST 1570a National Institute of Standards and Technology) and cabbage (IAEA-359 International Atomic Energy Agency Reference Materials Group), as reported in Table 4a,b.

### 2.4. Statistical Methods

Nutrient values were reported as the mean ± standard deviation of at least three determinations per sample. Data were expressed on a dry weight basis to account for potential dilution or concentration effects. The differences between the means were calculated using One-way Analysis of Variance (ANOVA) followed by the Bonferroni test, with a significance level of 0.05. Statistical analyses were performed using IBM SPSS Statistics 22 software (IBM, Armonk, NY, USA).

### 2.5. Percentage Change

Loss or increase change in mineral contents (on a dry weight basis) after cooking was expressed as a percentage and calculated by the following formula:

Percentage change % = [(xc − xr)/(xr) × 100]

The symbol xc indicates the nutrient content in cooked food, while xr the nutrient content in raw food.

### 2.6. Determination of Retention Factors

The true retention and apparent retention factors for the samples submitted to different cooking methods were calculated and expressed as percentages. As previously reported, the true retention factor (TR) accounts for the changes in solids of food during processing and cooking, whereas apparent retention factor (AR) provides a simplified measure of nutrient retention in foods after cooking and does not consider the losses of dry matter during processing [41,42].

#### 2.6.1. True Retention Factor

The TR % was determined experimentally by following the directions suggested by the USDA [42]. This method requires data regarding the weights of food before and after cooking, as well as the content of the nutrients in raw and cooked food. TR was calculated using the following equation [41,42]:

$$TR\%=\frac{nutrient\;content\;per\;g\;cooked\;food\;\left(fresh\;weight\;basis\right)\times g\;food\;after\;cooking}{nutrient\;content\;per\;g\;raw\;food\;\left(fresh\;weight\;basis\right)\times g\;food\;before\;cooking}\times 100$$

#### 2.6.2. Apparent Retention Factor

The AR was calculated on a moisture-free basis and using the following equation [41,42]:

$$AR\%=\frac{nutrient\;content\;per\;g\;cooked\;food\;(dry\;weight\;basis)\;}{nutrient\;content\;per\;g\;raw\;food\;(dry\;weight\;basis)}\times 100$$

## 3. Results

### 3.1. Proximate Composition

The macronutrient composition of the raw and cooked samples is reported in Table 5. The data indicate that the two zucchini varieties did not show significant changes in protein, dietary fiber, and starch (present in trace amounts) contents across the cooking methods, including steaming, microwaving, and grilling. A statistically significant change in simple sugars—which decreased from 43.1 g/100 g d.w. to 39.8 g/100 g d.w.—was observed in dark green zucchini after steaming. This variety also showed a significant change in total dietary fiber content, which increased after steaming and decreased after grilling. As for ash, in both types of zucchini, the content decreased significantly after cooking in the microwave and grilling. For eggplants, cooking methods did not cause significant changes in macronutrient content, except for grilling, which led to a significant increase in the simple sugar (from 38.8 g/100 g d. w. to 43.3 g/100 g d. w.). The heat treatment of potatoes by steaming and microwaving significantly affected their starch content, causing a decrease in starch content and a significant increase in soluble sugar content in response to microwave cooking.

### 3.2. Mineral Composition

#### 3.2.1. Romanesche Zucchini

The mineral composition of raw and cooked Romanesche zucchini is shown in Table 6. In the raw product, K (5382 mg/100 g d.w.) was found to be the most abundant macroelement, followed by P (892 mg/100 g), Mg (437 mg/100 g d.w.), Ca (296 mg/100 g d.w.), and Na (19 mg/100 g d.w.). The highest amount of trace element was Fe, followed by Zn and Mn. The different cooking methods did not lead to significant changes in the concentration of minerals, except for some combinations of cooking methods and minerals. For example, after grilling, the Ca content increased significantly, from 296 mg/100 g d.w. to 387 mg/100 g d.w., while it did not vary significantly after microwave cooking. On the contrary, the Na content changed significantly after microwave cooking, increasing from 19 mg/100 g d.w. to 24 mg/100 g d.w. This last cooking method also raised the Fe content.

The calculated retention factors are shown in Table 7. For steaming, the AR % values ranged from 81% to 117%, while the TR values ranged from 82% to 118%. For microwave cooking, the AR ranged from 96% to 124%, while the TR values ranged from 98% to 126%. After grilling, the calculated AR varied from 95% to 131% and the TR from 92% to 127%. When comparing AR and TR, the latter was slightly higher than AR for moist-heat cooking methods, whereas for grilling, this pattern was only observed for Mn.

#### 3.2.2. Dark Green Zucchini

The mineral content of dark green zucchini is presented in Table 8. In this variety of zucchini, the K was the most abundant macroelement detected (4309 mg/100 g d.w.), followed by P, Mg, Ca, and Na, all of which were determined in lower amounts than in the Romanesche variety. Conversely, trace elements were found in higher contents than in the Romanesche variety, with Fe being the most abundant (8.3 mg/100 g), followed by Zn (6.2 mg/100 g d.w.) and Mn (5.2 mg/100 g d.w.). Dark green zucchini did not undergo significant changes in mineral content, except for Na, which increased from 16 mg/100 g to 20 mg/100 g d.w. after grilling, and the Mn, which decreased after steaming and grilling. Microwave cooking did not appear to have significantly affected the content of any element. Overall, as with the Romanesche zucchini, the three cooking methods applied to this food did not substantially change the mineral profile compared to the raw product.

The percentage variations and retention factors calculated are reported in Table 9. In the case of steaming, the AR ranged from 78% to 108%, while the TR ranged between 92% and 104%. In the case of microwave cooking, the AR varied from 90% to 118%, while the TR spanned from 100% to 133%. For grilling, the AR was quite heterogeneous, with a range from 81% to 142%, while TR ranged from 77% to 134%.

#### 3.2.3. Eggplants

The mineral content values in raw and cooked eggplants are presented in Table 10. In the raw sample, K (2851 mg/100 g d.w.) was present in the highest concentration, followed by P, Ca, Mg, and Na. Among the trace elements, Fe (2.9 mg/100 g d.w.) was the most abundant, followed by Zn and Mn. As regards the impact of microwave cooking, the results of the ANOVA indicated that both thermal treatments resulted in notable differences in the concentration of various minerals. Microwave cooking has been found to significantly increase the values of Na, Mg, P, and Zn. Similarly, grilling was found to significantly improve the amount of Mg, P, and Mn, while simultaneously reducing the content of Na (from 38 to 36 mg/100 g d.w.) and Ca (from 222 to 158 mg/100 g d.w.). These values highlight that both cooking methods are optimal for sustaining the mineral quality profile of eggplants.

The heterogeneity of behavior is corroborated by the study of retention factors (Table 11), which revealed that the AR for microwave cooking ranged between 87% and 132%, while the TR ranged from 81% to 117%. Concerning grilling, the AR were relatively low, with a range from 71% to 110%, while the TR exhibited a value between 72% and 113%.

#### 3.2.4. Potatoes

The results presented in Table 12 confirmed that potatoes are an excellent source of minerals such as K (2232 mg/10 g d.w.), P (209 mg/100 g d.w.), and Mg (106 mg/100 g d.w.). However, it is important to note that the mineral content of potatoes can be influenced by various factors, such as the specific variety and postharvest conditions [43,44]. As demonstrated in Table 10, the cooking method employed (microwave and steaming) resulted in a notable reduction in the levels of various minerals analyzed, except for Mn. In detail, steaming significantly decreased the content of Na, Ca, Mg, Fe, and Zn, and similarly, microwave cooking reduced the amount of K, Ca, P, Fe, and Zn.

A comparison of the retention factors (Table 13) showed that the AR values for steaming varied from 76% to 106%, while the TR values ranged from 67% to 101%. In contrast, the AR values for microwave cooking varied from 78% to 103%, while the TR values ranged from 75% to 94%.

## 4. Discussion

The results of the analyses on two cultivars of zucchini, eggplant, and potatoes have allowed for a comparison of the effects of different home cooking methods on macronutrients and on minerals whose bibliography is particularly scarce.

### 4.1. Proximate Composition

Although each vegetable showed a distinct response, the statistical analysis demonstrated that heat treatments influenced the macronutrient content in different ways, including the carbohydrate profile (total dietary fiber, soluble sugars, and starch) and the ash content of dark green zucchini, eggplant, and potatoes. The different responses could be due to the combination of different cooking effects on these matrices. Cooking processes, in fact, are known to induce several structural changes in carbohydrates, potentially facilitating the conversion from one form to another [17,45]. According to Kumar and Aalbersberg [17], while the total carbohydrate content in foods remains largely unchanged, or varies only slightly, during cooking, the individual carbohydrate components frequently undergo significant alterations. Cooking vegetables produces a decrease in several fiber components due to the solubilization of polysaccharides which causes a decrease in total fiber content, mainly due to the loss of soluble fiber [46]. For example, as reported by Ur-Rehman et al. [45], cooking processes induce structural changes in the dietary fiber components of various vegetables by altering the ratio between soluble and insoluble fibers due to the reduction in neutral detergent fiber, acid detergent fiber, cellulose, hemicellulose, and lignin content. Moreover, some thermal treatments result in an increase in total dietary fiber, which is not attributed to new synthesis but rather to the formation of heat-resistant fiber–protein complexes that are measured as dietary fiber [46,47]. Even regarding starch and simple sugars, several concomitant phenomena can explain the different trends in the analyzed samples, making it difficult to deduce a generic effect of cooking on carbohydrates. In fact, cooking in humid heat can cause the absorption of water molecules in the amylose component of starch-rich foods, leaching them into the surrounding water [47]. Furthermore, Wei et al. [48] have demonstrated that cooking sweet potato storage roots decreased starch content and increased sugar concentration because starch is broken down by enzymes and converted into soluble sugars. At the same time, cooking involving water can facilitate the loss of sugars because of their high solubility [49]. This phenomenon could explain the significant increase in the soluble sugar content of potatoes, corresponding to the reduction in starch after microwave cooking, as reported in Table 5.

### 4.2. Minerals

As reported in Table 6, Table 8, Table 10 and Table 12, the mineral content was influenced in a heterogeneous manner by different household cooking methods. Regarding the two varieties of zucchini, these samples exhibited less pronounced effects in terms of mineral content variation than the other two kinds of vegetables. Furthermore, despite the two zucchini varieties being prepared in the same way and cooked using the same methods and for similar times, they exhibited different variations in mineral content. This indicates that the intrinsic characteristics of food influence mineral retention or loss. A study published by de Castro et al. [29] revealed that the mineral profile of Brazilian zucchini, carrots, and broccoli is influenced by cooking methods in different ways, not only across different plant species, but also in response to the type of cultivation.

The eggplants demonstrated discrepancies in their response to cooking techniques, particularly in microwave cooking, which resulted in a significant increase in Na, Mg, P, and Zn. It is possible that the contact of the food with tap water during microwave cooking (Table 1) could increase the mineral content, as evident in some minerals of zucchini and eggplants.

In contrast, potatoes exhibited a notable alteration in their mineral content, with a decline observed in the levels of many minerals after both cooking methods were applied, maybe due to the destruction of the potato microstructure [47]. As reported by Coe [50], in potatoes, the decrease in mineral content is low when cooking is carried out with dry-heat methods as opposed to moist-heat methods, which can cause the loss of minerals, mainly K, P, and Mg. The discrepancy in the cooking responses of potatoes in comparison to other vegetables can be attributed to a combination of their inherent characteristics and the preparation process, which involves peeling and chopping them into smaller pieces, and which would have made them more susceptible to mineral loss by increasing their surface area exposed to moist heat. This is supported by the negligible loss of Fe and Zn when whole unpeeled potatoes are boiled [51]. On the contrary, the cutting of vegetables into large cylindrical shapes has been demonstrated to largely preserve their nutritional qualities, limiting the loss of micronutrients [28,52].

As reported by Bethke and Jansky [53], the K and other mineral contents were significantly reduced (75%) if the samples were shredded prior to boiling, and by 45% if the samples were cubed prior to boiling. Furthermore, the mineral content may be influenced by certain variability in the thickness of the peel cut, which has a different mineral concentration from the pulp [25,44]. It is therefore advisable to cook the potatoes with the peel on and cut into large pieces to limit the loss of minerals.

Overall, the mineral profile alterations occur in different patterns, and the data indicate that the extent of nutritional variation depends on the nature of the food product and the nutrients, the food preparation procedures, and the cooking method and timing. Indeed, minerals can exist in foods in different forms within plant tissues, for example, Ca, K, and Fe are bonded with high-molecular-weight compounds such as proteins, thereby impeding their solubilization [4,21].

Furthermore, mineral compartmentalization within plant tissues may also influence heat response behavior. Since Na is predominantly an extracellular ion, it is susceptible to leaching during water-based treatment protocols. In contrast, K, an intracellular ion, is present in lowest concentrations in the extracellular space. However, it can also be released into the external environment following heat-induced cell wall loosening [29].

#### Dietary Consideration: Focus on Fe and Zn

Understanding the mineral content of foods is essential for determining the optimal cooking methods that can help maintain their nutritional quality, particularly for trace elements like Fe and Zn, which are critical in addressing global health issues among vulnerable populations, such as women and the elderly [54]. Zn deficiency can lead to serious health problems, including the loss of appetite, skin lesions, impairments in taste and smell, and alterations in the metabolism of carbohydrates and proteins [54,55]. Fe deficiency mainly causes anemia, affects cognitive functions and physical energy, and presents symptoms such as fatigue and memory difficulties [55].

Considering the Fe content in a standard portion (200 g) of “fresh weight” samples, Romanesche zucchini and dark green zucchini cooked in a non-stick pan were the samples that were the closet to meeting the Population Recommended Intake (PRI) of Fe for adult men (10 mg daily) and women (18 mg daily), as suggested by the Dietary Reference Values for Nutrients and Energy for the Italian population [56]. I fact, a serving of Romanesche zucchini contributes to 17% (for men) and 9% (for women) of the recommended intake, and dark green zucchini provides similar values corresponding to 18% and 10% of PRI of Fe [56]. These values are higher compared to raw zucchini or those cooked in a microwave or steamed, since grilling resulted in a significant water loss, thus concentrating the nutrients. In comparison, eggplants and potatoes, which inherently contain less Fe, contribute less to the recommended intake. Furthermore, eggplants grilled in a non-stick pan provided a greater quantity of Fe per fresh weight compared to those cooked in a microwave, covering 6% and 4% of the PRI for men and women, respectively. A portion of potatoes cooked in a steamer or in the microwave, on the other hand, contributes in a similar way to the PRI, with a value of about 6% for men and 3% for women.

As regards Zn, grilled zucchini has the highest content per fresh product, and a 200 g serving of both Romanesche and dark green zucchini provides around 12% and 16% of the PRI for adult men (12 mg/day) and women (9 mg/day), respectively [56]. In contrast, grilled eggplant provides 3% of the PRI coverage for men and 4% for women, while microwaved eggplant provides about 2% and 3%, respectively. For potatoes, a microwaved serving provides 5% of the recommended daily amount for men and 6% for women, values slightly higher than the steamed one (4% and 5%).

### 4.3. Retention Factors

The calculated retention factors (Table 7, Table 9, Table 11 and Table 13) included in this study reflect the heterogeneous trend of minerals after cooking. The results demonstrate that for zucchini varieties, the TR factors of steaming and grilling are lower than those of microwave cooking, except for some minerals. On the contrary, for eggplant, the TR of grilling tends to be higher than that obtained after microwave. Similar findings were reported by Lee et al. [7], who observed that the TR of minerals were higher for mushrooms subjected to microwave cooking and grilling than for those treated with steaming, blanching, and boiling. The high values (TR > 100) found in some minerals can be explained in different ways, for example, contact with tap water during washing and cooking, especially in the case of microwave cooking; contact with cookware; and the possible loss of solid portions such as fiber, that can cause a concentration of other nutrients such as minerals [5,21,33,57]. These conditions could have occurred, especially in the case of grilling, which involves a lot of handling and turning of the product over the pan. Furthermore, the variability and analytical error must be considered.

The lack of experimental data on mineral loss makes result comparisons with other published research difficult. The scarcity of available data on retention factors is a consequence of the inherent difficulty in conducting studies on such a vast number of food-cooking combinations. It should be noted that the TR values present in some composition tables and reference documents often do not refer to a single food item, but rather to an entire food group or subgroup that has undergone heat treatment. To illustrate this, the document published by Bell et al. [22] contains TR data reported for the generic category “vegetables” subject to frying, boiling, and baking. In this paper, the TR values for Na, K, Mg, C, P, and Fe are reported as 100%, but they do not account for the differences between products. Indeed, the existing bibliography is often limited to references to traditional cooking procedures—that are carried out in different ways and with varying preparation times in different countries—making it challenging to draw comparisons. Similar condition can be found in the report published by Vásquez-Caicedo [58]. One of the most cited reference papers is the report published by Bognar [30], which contains the TR values for different nutrients for a group of vegetables that includes 12 different foods, comprising zucchini and eggplant. These vegetables, subjected to steaming, showed TR values for minerals that ranged from 80% to 100% [30]. The same document reports a TR for the category of potatoes, which also includes potato-based dishes. The TR of minerals for peeled potatoes subjected to steaming range from 85% (for K) to 95% (all other minerals). Additionally, the document published by the USDA in 2007 reports TR values for minerals that are approximately 90–95% for moist steam cooking, such as boiling for potato with skin, and 100% for a generic group of vegetables [42].

As regards the discrepancy between the TR% and AR% found for a given mineral in each sample, this depends on the specific cooking method and the nature of the foods and is determined by the quantity of water lost or gained during cooking. As Murphy [33] has observed, it is not possible to predict the significance or magnitude of the differences between TR and AR.

Consequently, it is not feasible to establish correction factors which could be applied to AR to estimate TR. This condition emphasizes the necessity for foods to be weighed with precision, both prior to and following the cooking process, in studies that examine composition to determine the real retention factor.

## 5. Conclusions

Quality data on the nutritional composition of cooked foods are essential for health and nutrition professionals, as well as for the food industry and consumers, as it guides the selection of optimal cooking methods that help preserve the nutritional value of foods. This study highlights the complex interactions between cooking methods and the macronutrient and mineral content in vegetables like zucchinis, eggplants, and potatoes. The results indicate that heat treatments significantly impact carbohydrate profiles and mineral content, varying widely between vegetables due to intrinsic factors and cooking techniques. Notably, zucchini showed a relatively stable mineral content across cooking methods, while eggplants exhibited increased mineral levels after grilling and microwave cooking. In contrast, cooking methods significantly reduced mineral levels in potatoes, highlighting the impact of peeling and cutting on structural changes during cooking.

From a more strictly nutritional point of view and considering the product “as consumed”, the two kinds of zucchini cooked in a non-stick pan provide substantial contributions to the recommended daily intakes of Fe and Zn, surpassing other cooking methods that lose less water.

Our research also underscores the intricacies of mineral retention factors, attributed to food type, preparation techniques, and the potential for leaching during cooking processes. Moreover, the considerable discrepancy between the “true” and “apparent” retention factors, which are influenced by the variation in water content during cooking, highlights the necessity for further experimental data that can enhance the existing bibliographic references which are currently lacking and outdated.

## Figures and Tables

**Table 1:** Preparation and cooking method applied to the samples.

Sample	Food Preparation	Cooking Method	Timing (min.)	Weight After Cooking/Weight Before Cooking
Romanesche Zucchini	Unpeeled, whole	Steaming	30 ± 0.0	0.9 ± 0.01
	Unpeeled, whole + 125 mL of tap water, covered with cling film	Microwave	13 ± 0.0	0.8 ± 0.03
	Unpeeled, longitudinal slices	Grilling	16 ± 5.7	0.4 ± 0.05
Dark green Zucchini	Unpeeled, whole	Steaming	30 ± 0.0	0.9 ± 0.01
	Unpeeled, whole + 200 mL of tap water, covered with cling film	Microwave	14 ± 1.4	0.9 ± 0.03
	Longitudinal slices	Grilling	20 ± 0.7	0.5 ± 0.09
Eggplant	Unpeeled, cut in cubes + 170 mL of tap water, covered with cling film	Microwave	16 ± 1.4	0.9 ± 0.09
	Unpeeled, cut in transversal slices	Grilling	14 ± 1.4	0.6 ± 0.08
Potato	Peeled, cut into cubes	Steaming	25 ± 0.0	1.0 ± 0.01
	Peeled, cut into cubes + 125 mL of tap water, covered with cling film	Microwave	20 ± 0.0	0.8 ± 0.08

Modified from Lisciani et al., 2022 [36].

**Table 2:** Mineral composition of tap water in Rome.

Minerals	mg/L
Na	7.3 ± 0.0
K	13. 9 ± 0.0
Ca	74.3 ± 0.0
Mg	15.0 ± 0.0
P	0.85 ± 0.01
Fe	0.10 ± 0.00
Zn	0.84 ± 0.01
Mn	0.00 ± 0.00

Data are expressed as the mean ± standard deviation.

**Table 3:** ICP-OES instrument operating parameters.

Method Parameter	Values
Plasma view	Axial/Attenuate axial
View distance	15 mm
Plasma gas flow	10 L/min
Auxiliary gas flow	0.2 L/min
Pump flow rate	1.00 mL/min
Detector	Dual backside-illuminated charge-coupled device.
Power	1500 watt
Nebulizer	0.65 L/min
Read	Peak area

**Table 4:** (**a**) NIST 1570 spinach certified values, measured values and % recovery. (**b**) IEAE 359 cabbage certified values, measured values and % recovery.

(**a**)			
**Minerals**	**Certified Values (mg/100)**	**Measured Values (mg/100)**	**Recovery (%)**
Na	1818.0	1846.6 ± 92.43	101.6
K	2903.0	2897.7 ± 102.37	99.8
Ca	1527.0	1445.8 ± 52.23	94.7
P	518.0	510.3 ± 17.52	98.5
Zn	8.2	7.5 ± 0.17	91.6
Mn	7.6	7.0 ± 0.22	92.3
(**b**)			
Na	58.0	49.8 ± 1.87	85.8
K	3250.0	2998.8 ± 310.86	92.3
Ca	1850.0	1834.1 ± 11.76	99.1
Mg	216.0	204.5 ± 23.71	94.7
Fe	14.8	12.3 ± 0.32	82.9
Zn	3.9	3.5 ± 0.52	90.4
Mn	3.2	2.9 ± 0.42	91.4

Measured data are expressed as the mean ± standard deviation.

**Table 5:** Proximate composition of studied vegetables before and after different cooking methods (g/100 g d.w.).

Sample		Protein	Lipid	Total Starch	Soluble Sugars	Total Dietary Fiber	Ash
Romanesche Zucchini	Raw	30.0 ± 4.29 ^a^	1.5 ± 0.10 ^a^	tr *	36.2 ± 2.05 ^a^	19.5 ± 0.35 ^a^	13.7 ± 0.68 ^a^
	Steam	28.3 ± 0.44 ^a^	1.4 ± 0.08 ^a^	*tr*	35.2 ± 2.00 ^a^	19.0 ± 0.75 ^a^	16.9 ± 0.43 ^a^
	Microwave	29.1 ± 0.39 ^a^	1.4 ± 0.07 ^a^	*tr*	37.0 ± 1.22 ^a^	20.1 ± 0.11 ^a^	12.7 ± 0.00 ^b^
	Grill	30.4 ± 0.58 ^a^	1.3 ± 0.10 ^a^	*tr*	37.2 ± 0.55 ^a^	18.5 ± 1.01 ^a^	12.7 ± 0.19 ^b^
Dark green Zucchini	Raw	22.2 ± 0.40 ^a^	1.6 ± 0.16 ^a^	*tr*	43.1 ± 0.99 ^a^	18.9 ± 0. 20 ^a^	14.4 ± 0.20 ^a^
	Steam	23.3 ± 0.37 ^a^	1.4 ± 0.26 ^a^	*tr*	39.8 ± 1.10 ^b^	21.4 ± 1.06 ^c^	13.8 ± 0.00 ^a,b^
	Microwave	22.3 ± 0.50 ^a^	1.5 ± 0.04 ^a^	*tr*	44.3 ± 0.09 ^a^	18.4 ± 0.45 ^a,b^	13.2 ± 0.71 ^b^
	Grill	22.5 ± 0.52 ^a^	1.5 ± 0.05 ^a^	*tr*	47.1 ± 0.22 ^a^	17.1 ± 0.40 ^b^	11.2 ± 0.10 ^c^
Eggplants	Raw	12.8 ± 0.80 ^a^	6.2 ± 0.09 ^a^	*tr*	38.8 ± 1.10 ^a^	33.7 ± 1.55 ^a^	8.7 ± 0.09 ^a^
	Microwave	11.9 ± 0.17 ^a^	5.8 ± 0.16 ^a^	*tr*	39.2 ± 0.67 ^a^	34.0 ± 1.90 ^a^	8.0 ± 0.15 ^b^
	Grill	11.6 ± 0.55 ^a^	5.8 ± 0.05 ^a^	*tr*	43.3 ± 1.04 ^b^	33.2 ± 1.53 ^a^	8.1 ± 0.14 ^b^
Potatoes	Raw	12.5 ± 0.89 ^a^	4.8 ± 0.01 ^a^	76.6 ± 0.25 ^a^	1.1 ± 0.28 ^a^	7.9 ± 0.15 ^a^	4.9 ± 0.89 ^a^
	Steam	10.7 ± 1.46 ^a^	5.1 ± 0.09 ^a^	71.1 ± 3.04 ^b^	1.3 ± 0.12 ^a^	7.9 ± 0.19 ^a^	5.4 ±0.70 ^a^
	Microwave	10.9 ± 0.32 ^a^	4.8 ± 0.03 ^a^	64.5 ± 1.22 ^c^	5.3 ± 0.12 ^b^	7.7 ± 0.94 ^a^	5.6 ± 0.53 ^a^

Data are expressed as the mean ± standard deviation. Anova, Bonferroni Test: by column, the means with different letters are significantly different at *p* < 0.05. * *tr* ≤ limit of detection (0.19 g/100 g).

**Table 6:** Levels of minerals (mg/100 g d.w.) in raw and cooked Romanesche zucchini.

Minerals	Raw	Steam	Microwave	Grill
Na	19 ± 1.7 ^a,b,c^	16 ± 0.5 ^b^	24 ± 0.2 ^c^	19 ± 0.47 ^a^
K	5382 ± 220.0 ^a^	4974 ± 89.0 ^a^	5247 ± 76.0 ^a^	6710 ± 45.4 ^b^
Ca	296 ± 0.9 ^a^	240 ± 3.2 ^b^	283 ± 1.3 ^a^	387 ± 8.7 ^c^
Mg	437 ± 4.9 ^a^	428 ± 3.0 ^a^	451 ± 4.2 ^a^	433 ± 8.5 ^a^
P	892 ± 33.1 ^a^	865 ± 0.8 ^a^	922 ± 4.7 ^a^	848 ± 13.2 ^a^
Fe	5.7 ± 0.16 ^a^	6.7 ± 0.32 ^a,b^	6.7 ± 0.03 ^b^	5.5 ± 0.19 ^a^
Zn	5.0 ± 0.05 ^a^	5.0 ± 0.08 ^a^	5.0 ± 0.03 ^a^	4.9 ± 0.14 ^a^
Mn	1.9 ± 0.01 ^a^	1.9 ± 0.03 ^a^	2.0 ± 0.00 ^a^	2.0 ± 0.04 ^a^

Data are expressed as the mean ± standard deviation. Anova, Bonferroni Test: by row, the means followed by different letters are significantly different at *p* < 0.05.

**Table 7:** Percentage change, apparent retention factors (AR), and true retention factors (TR) for minerals in Romanesche zucchini.

Minerals	Steam			Microwave			Grill		
	Percentage Change (%)	AR (%)	TR (%)	Percentage Change (%)	AR (%)	TR (%)	Percentage Change (%)	AR (%)	TR (%)
Na	−17	83	84	24	124	126	−2	99	97
K	−8	92	93	−2	98	100	25	125	121
Ca	−19	81	82	−4	96	98	31	131	127
Mg	−2	98	99	3	103	105	−1	99	96
P	−3	97	98	3	103	106	−5	95	92
Fe	17	117	118	17	117	121	−3	97	95
Zn	−1	99	99	−1	99	101	−3	97	94
Mn	3	103	106	6	106	109	5	105	106

**Table 8:** Levels of minerals (mg/100 g d.w.) in raw and cooked dark green zucchini.

Minerals	Raw	Steam	Microwave	Grill
Na	14 ± 1.3 ^a^	14 ± 0.0 ^a^	16 ± 0.7 ^a,b^	20 ± 1.3 ^b^
K	4309 ± 76.9 ^a^	4070 ± 43.3 ^a^	4103 ± 0.5 ^a^	4101 ± 320.0 ^a^
Ca	234 ± 8.8 ^a^	245 ± 0.6 ^a^	258 ± 2.3 ^a^	244 ± 14.6 ^a^
Mg	367 ± 16.1 ^a^	360 ± 0.9 ^a^	347 ± 0.0 ^a^	363 ± 24.7 ^a^
P	696 ± 27.0 ^a^	712 ± 2.0 ^a^	688 ± 13.7 ^a^	701 ± 40.8 ^a^
Fe	8.3 ± 0.63 ^a^	8.9 ± 0.27 ^a^	7.4 ± 0.01 ^a^	8.0 ± 0.45 ^a^
Zn	6.2 ± 0.18 ^a^	6.1 ± 0.02 ^a^	5.8 ± 0.09 ^a^	5.9 ± 0.17 ^a^
Mn	5.2 ± 0.21 ^a^	4.1 ± 0.04 ^b^	4.8 ± 0.07 ^a,b^	4.2 ± 0.34 ^b^

Data are expressed as the mean ± standard deviation. Anova, Bonferroni Test: by row, the means followed by different letters are significantly different at *p* < 0.05.

**Table 9:** Percentage change, apparent retention factors (AR), and true retention factors (TR) for minerals in dark green zucchini.

Minerals	Steam			Microwave			Grill		
	Percentage Change (%)	AR (%)	TR (%)	Percentage Change (%)	AR (%)	TR (%)	Percentage Change (%)	AR (%)	TR (%)
Na	1	101	99	18	118	133	42	142	134
K	−8	92	92	−7	93	108	−7	93	90
Ca	5	105	103	11	111	125	4	104	99
Mg	−2	98	96	−6	94	107	−1	99	93
P	2	102	100	−1	99	112	1	101	95
Fe	8	108	104	−10	90	100	−7	93	87
Zn	−2	98	97	−5	95	108	−5	95	90
Mn	−22	78	76	−8	92	104	−19	81	77

**Table 10:** Levels of minerals (mg/100 g d.w.) in raw and cooked eggplants.

Minerals	Raw	Microwave	Grill
Na	38 ± 0.1 ^a^	39 ± 0.0 ^b^	36 ± 0.2 ^c^
K	2851 ± 106.9 ^a^	3095 ± 32.4 ^a^	2977 ± 17.3 ^a^
Ca	222 ± 2.5 ^a^	228 ± 0.8 ^a^	158 ± 0.5 ^b^
Mg	198 ± 0.5 ^a^	212 ± 2.3 ^b^	209 ± 4.1 ^b^
P	297 ± 1.5 ^a^	321 ± 2.2 ^b^	326 ± 4.6 ^b^
Fe	2.9 ± 0.04 ^a^	2.5 ± 0.00 ^a^	2.8 ± 0.03 ^a^
Zn	1.5 ± 0.03 ^a^	2.0 ± 0.07 ^b^	1.6 ± 0.02 ^a^
Mn	1.4 ± 0.00 ^a^	1.4 ± 0.01 ^a^	1.6 ± 0.03 ^b^

Data are expressed as the mean ± standard deviation. Anova, Bonferroni Test: by row, the means followed by different letters are significantly different at *p* < 0.05.

**Table 11:** Percentage change, apparent retention factors (AR), and true retention factors (TR) for minerals in eggplants.

Minerals	Microwave			Grill		
	Percentage Change (%)	AR (%)	TR (%)	Percentage Change (%)	AR (%)	TR (%)
Na	4	104	96	−5	95	96
K	9	109	101	4	104	105
Ca	3	103	96	−29	71	72
Mg	7	107	100	6	106	107
P	8	108	101	10	110	111
Fe	−13	87	81	−2	98	101
Zn	32	132	117	7	107	108
Mn	−3	97	90	10	110	113

**Table 12:** Levels of minerals (mg/100 g d.w.) in raw and cooked potatoes.

Minerals	Raw	Steam	Microwave
Na	11 ± 1.5 ^a^	8 ± 0.2 ^b^	10 ± 0.7 ^a^
K	2232 ± 86.3 ^a^	2102 ± 58.6 ^a,b^	2008 ± 102.3 ^b^
Ca	38 ± 1.0 ^a^	31 ± 0.6 ^b^	33 ± 1.3 ^b^
Mg	106 ± 1.6 ^a^	100 ± 2.2 ^b^	108 ± 1.3 ^a^
P	209 ± 6.5 ^a,b^	222 ± 6.5 ^a^	196 ± 9.5 ^b^
Fe	1.6 ± 0.04 ^a^	1.4 ± 0.03 ^c^	1.3 ± 0.04 ^b^
Zn	1.38 ± 0.05 ^a^	1.22 ± 0.05 ^b^	1.17 ± 0.13 ^b^
Mn	0.41 ± 0.01 ^a^	0.40 ± 0.01 ^a^	0.42 ± 0.02 ^a^

Data are expressed as the mean ± standard deviation. Anova, Bonferroni Test: by row, the means followed by different letters are significantly different at *p* < 0.05.

**Table 13:** Variation percentage, apparent retention factors (AR), and true retention factors (TR) for minerals in potatoes.

Minerals	Steam			Microwave		
	Percentage Change (%)	AR (%)	TR (%)	Percentage Change (%)	AR (%)	TR (%)
Na	−24	76	67	−5	95	88
K	−6	94	89	−10	90	83
Ca	−18	82	78	−13	87	80
Mg	−6	94	89	1	101	94
P	6	106	101	−6	94	87
Fe	−16	84	81	−22	78	75
Zn	−11	89	83	−15	85	77
Mn	−2	98	89	3	103	89

## Data Availability

The data supporting the conclusions of this article will be made available by the authors on request.

## References

[1] Rees K., Takeda A., Martin N., Ellis L., Wijesekara D., Vepa A., Das A., Hartley L., Stranges S. (2020). Mediterranean-Style Diet for the Primary and Secondary Prevention of Cardiovascular Disease: A Cochrane Review. Glob. Heart.

[2] Minihane A.M., Murphy K.J. (2022). The Health Benefits and Practical Considerations for the Adoption of a Mediterranean-Style Dietary Pattern. Br. J. Nutr..

[3] Madsen H., Sen A., Aune D. (2023). Fruit and Vegetable Consumption and the Risk of Hypertension: A Systematic Review and Meta-Analysis of Prospective Studies. Eur. J. Nutr..

[4] García-Herrera P., Morales P., Cámara M., Fernández-Ruiz V., Tardío J., Sánchez-Mata M.C. (2020). Nutritional and Phytochemical Composition of Mediterranean Wild Vegetables after Culinary Treatment. Foods.

[5] Fratianni A., D’Agostino A., Niro S., Panfili G., Bufano A., Paura B. (2021). Loss or Gain of Lipophilic Bioactive Compounds in Vegetables after Domestic Cooking? Effect of Steaming and Boiling. Foods.

[6] Fratianni A., Albanese D., Ianiri G., Vitone C., Malvano F., Avino P., Panfili G. (2024). Evaluation of the Content of Minerals, B-Group Vitamins, Tocols, and Carotenoids in Raw and In-House Cooked Wild Edible Plants. Foods.

[7] Lee K., Lee H., Choi Y., Kim Y., Jeong H.S., Lee J. (2019). Effect of Different Cooking Methods on the True Retention of Vitamins, Minerals, and Bioactive Compounds in Shiitake Mushrooms (*Lentinula edodes*). Food Sci. Technol. Res..

[8] Miglio C., Chiavaro E., Visconti A., Fogliano V., Pellegrini N. (2008). Effects of Different Cooking Methods on Nutritional and Physicochemical Characteristics of Selected Vegetables. J. Agric. Food Chem..

[9] Crosby G. (2024). The Key Role of Cooking and Food Preparation in Affecting Nutrient Composition of Foods. Precis. Nutr. Sci. Promise Pers. Nutr. Health.

[10] Bureau S., Mouhoubi S., Touloumet L., Garcia C., Moreau F., Bédouet V., Renard C.M.G.C. (2015). Are Folates, Carotenoids and Vitamin C Affected by Cooking? Four Domestic Procedures Are Compared on a Large Diversity of Frozen Vegetables. LWT.

[11] Landry M.J., Burgermaster M., van den Berg A.E., Asigbee F.M., Vandyousefi S., Ghaddar R., Jeans M.R., Yau A., Davis J.N. (2020). Barriers to Preparing and Cooking Vegetables Are Associated with Decreased Home Availability of Vegetables in Low-Income Households. Nutrients.

[12] Czarnowska-Kujawska M., Draszanowska A., Chróst M., Starowicz M. (2023). The Effect of Sous-Vide Processing Time on Chemical and Sensory Properties of Broccoli, Green Beans and Beetroots. Appl. Sci..

[13] Douiri-Bedoui I., Abdellaoui H., Alexa R., Jacolot P., Druon C., Tessier F.J., Laguerre J.-C. (2011). Optimization of Microwave Cooking of Courgette in Terms of Nutrient Preservation and Energy Consumption. Procedia Food Sci..

[14] Zhong X., Dolan K.D., Almenar E. (2015). Effect of Steamable Bag Microwaving versus Traditional Cooking Methods on Nutritional Preservation and Physical Properties of Frozen Vegetables: A Case Study on Broccoli (*Brassica oleracea*). Innov. Food Sci. Emerg. Technol..

[15] Fabbri A.D.T., Crosby G.A. (2016). A Review of the Impact of Preparation and Cooking on the Nutritional Quality of Vegetables and Legumes. Int. J. Gastron. Food Sci..

[16] Bergstrom L. (1994). Rapport 32/94: Nutrient Losses and Gains in the Preparation of Foods.

[17] Kumar S., Aalbersberg B. (2006). Nutrient Retention in Foods after Earth-Oven Cooking Compared to Other Forms of Domestic Cooking. 2. Vitamins. J. Food Compos. Anal..

[18] Razzak A., Mahjabin T., Khan M.R.M., Hossain M., Sadia U., Zzaman W. (2023). Effect of Cooking Methods on the Nutritional Quality of Selected Vegetables at Sylhet City. Heliyon.

[19] Alajaji S.A., El-Adawy T.A. (2006). Nutritional Composition of Chickpea (*Cicer arietinum* L.) as Affected by Microwave Cooking and Other Traditional Cooking Methods. J. Food Compos. Anal..

[20] Yuan X., Fujiwara A., Matsumoto M., Tajima R., Shinsugi C., Koshida E., Takimoto H. (2022). Definitions and Assessment Methods of ‘Home Cooking’ in Studies with Dietary Variables: A Scoping Review. Nutrients.

[21] Huey S.L., Konieczynski E.M., Mehta N.H., Krisher J.T., Bhargava A., Friesen V.M., Mbuya M.N.N., Monterrosa E.C., Nyangaresi A.M., Mehta S. (2023). A Systematic Review of the Impacts of Post-Harvest Handling on Provitamin A, Iron and Zinc Retention in Seven Biofortified Crops. Nat. Food.

[22] Bell S., Becker W., Vásquez-Caicedo, Hartmann A.L. (2006). European food information resource network (EuroFIR) Workpackage1.5 Standards Development. Report on Nutrient Losses and Gains Factors Used in European Food Composition Databases D 1.5.5-NLG-Factors-Inventory.

[23] Bernhardt S., Schlich E. (2006). Impact of Different Cooking Methods on Food Quality: Retention of Lipophilic Vitamins in Fresh and Frozen Vegetables. J. Food Eng..

[24] Onyeka U.E., Ibeawuchi O.N. (2021). Loss of food nutrients orchestrated by cooking pots: A common trend in developing world. J. Food Sci. Technol..

[25] Subramanian N.K., White P.J., Broadley M.R., Ramsay G. (2011). The Three-Dimensional Distribution of Minerals in Potato Tubers. Ann. Bot..

[26] Kumar A., Gupta K., Islam Apu M.A., Abrol G.S., Tomer V. (2023). Effect of Household Processing on Nutritional and Antinutritional Composition, Mineral-Mineral Ratios, and Functional Properties of Colocasia Leaves. Heliyon.

[27] Lachman J., Hamouz K., Musilová J., Hejtmánková K., Kotíková Z., Pazderů K., Domkářová J., Pivec V., Cimr J. (2013). Effectofpeeling and three cooking methods on the content of selected phytochemicals in potato tubers with various colour of flesh. Food Chem..

[28] Gelaye Y. (2023). Quality and Nutrient Loss in the Cooking Vegetable and Its Implications for Food and Nutrition Security in Ethiopia: A Review. Nutr. Diet. Suppl..

[29] De Castro N.T., de Alencar E.R., Zandonadi R.P., Han H., Raposo A., Ariza-Montes A., Araya-Castillo L., Botelho R.B.A. (2021). Influence of Cooking Method on the Nutritional Quality of Organic and Conventional Brazilian Vegetables: A Study on Sodium, Potassium, and Carotenoids. Foods.

[30] Bognár A., Piekarski J. (2000). Guidelines for Recipe Information and Calculation of Nutrient Composition of Prepared Foods (Dishes). J. Food Compos. Anal..

[31] Lešková E., Kubíková J., Kováčiková E., Košická M., Porubská J., Holčíková K. (2006). Vitamin Losses: Retention during Heat Treatment and Continual Changes Expressed by Mathematical Models. J. Food Compos. Anal..

[32] Showell B.A., Howe J.C., Williams J.R., Holden J.M., Zeisel S. (2007). Determination of cooking yields and nutrient retention factors of choline in meat products. Exp. Biol..

[33] Murphy E.W., Criner P.E., Gray B.C. (1975). Comparisons of Methods for Calculating Retentions of Nutrients in Cooked Foods. J. Agric. Food Chem..

[34] Badiani A., Stipa S., Bitossi F., Pirini M., Bonaldo A., Gatta P.P., Rotolo M., Testi S. (2013). True retention of nutrients upon household cooking of farmed portion-size European sea bass (*Dicentrarchus labrax* L.). LWT-Food Sci. Technol..

[35] Wu X., Zhao Y., Haytowitz D.B., Chen P., Pehrsson P.R. (2019). Effects of Domestic Cooking on Flavonoids in Broccoli and Calculation of Retention Factors. Heliyon.

[36] Lisciani S., Camilli E., Marletta L., Marconi S. (2022). Weight Change of Food after Cooking: Focus on the Italian Food Composition Tables Appendix. Int. J. Gastron. Food Sci..

[37] AOAC International (2023). Official Methods of Analysis of AOAC International.

[38] Thiex N.J., Anderson S., Gildemeister B. (2023). Crude fat, hexanes extraction, in feed, cereal grain, and forage (Randall/Soxtec/submersion method): Collaborative study. J. AOAC Int..

[39] McCleary B.V. (2023). Measurement of Dietary Fiber: Which AOAC Official Method of AnalysisSM to Use. J. AOAC Int..

[40] McCleary B.V., Charmier L.M.J., McKie V.A. (2019). Measurement of Starch: Critical Evaluation of Current Methodology. Starch/Staerke.

[41] Hummel M., Talsma E.F., Taleon V., Londoño L., Brychkova G., Gallego S., Raatz B., Spillane C. (2020). Iron, Zinc and Phytic Acid Retention of Biofortified, Low Phytic Acid, and Conventional Bean Varieties When Preparing Common Household Recipes. Nutrients.

[42] USDA (2007). Table of Nutrient Retention Factors. https://catalog.data.gov/dataset/usda-table-of-nutrient-retention-factors-release-6-2007-1b152.

[43] Burlingame B., Mouillé B., Charrondière R. (2009). Nutrients, Bioactive Non-Nutrients and Anti-Nutrients in Potatoes. J. Food Compos. Anal..

[44] Bnv P., Gvs S. (2023). Potato—Powerhouse for Many Nutrients. Potato Res..

[45] Ur-Rehman Z., Islam M., Shah W.H. (2003). Effect of Microwave and Conventional Cooking on Insoluble Dietary Fibre Components of Vegetables. Food Chem..

[46] Dhingra D., Michael M., Rajput H., Patil R.T. (2012). Dietary Fibre in Foods: A Review. J. Food Sci. Technol..

[47] Jayanty S.S., Diganta K., Raven B. (2019). Effects of Cooking Methods on Nutritional Content in Potato Tubers. Am. J. Potato Res..

[48] Wei S., Lu G., Cao H. (2017). Effects of Cooking Methods on Starch and Sugar Composition of Sweetpotato Storage Roots. PLoS ONE.

[49] Kumar S., Aalbersberg B. (2006). Nutrient Retention in Foods after Earth-Oven Cooking Compared to Other Forms of Domestic Cooking. 1. Proximates, Carbohydrates and Dietary Fibre. J. Food Compos. Anal..

[50] Coe S., Spiro A. (2022). Cooking at Home to Retain Nutritional Quality and Minimise Nutrient Losses: A Focus on Vegetables, Potatoes and Pulses. Nutr. Bull..

[51] Burgos G., Amoros W., Morote M., Stangoulis J., Bonierbale M. (2007). Iron and Zinc Concentration of Native Andean Potato Cultivars from a Human Nutrition Perspective. J. Sci. Food Agric..

[52] Kapusta-Duch J., Florkiewicz A., Leszczyńska T., Borczak B. (2021). Directions of Changes in the Content of Selected Macro-and Micronutrients of Kale, Rutabaga, Green and Purple Cauliflower Due to Hydrothermal Treatment. Appl. Sci..

[53] Bethke P.C., Jansky S.H. (2008). The Effects of Boiling and Leaching on the Content of Potassium and Other Minerals in Potatoes. J. Food Sci..

[54] Mehri A. (2020). Trace elements in human nutrition (II)—An update. Int. J. Prev. Med..

[55] Poonam S., Surendra P. (2023). A review on iron, zinc and calcium biological significance and factors affecting their absorption and bioavailability. J. Food Compos. Anal..

[56] SINU, Società Italiana di Nutrizione Umana (2024). Livelli di Assunzione di Riferimento di Nutrienti ed Energia per la Popolazione Italiana.

[57] Williams-Ngegba M.S.E., Onabanjo O.O., Anthony N.M., Alamu E.O., Maziya-Dixon B., Oguntona E.B. (2024). Variations in Micronutrient Concentrations and Retentions in Fufu Made from Yellow-Fleshed Cassava as a Function of Genotype and Processing Methods. Front. Nutr..

[58] Vasquez-Caicedo A.L., Bell S., Hartmann B. (2008). Eurofir. Report on Recipe Calculation Procedures for Composite Foods. https://www.eurofir.org/report-on-collection-of-rules-on-use-of-recipe-calculation-procedures-including-the-use-of-yield-and-retention-factors-for-imputing-nutrient-values-for-composite-foods/.

